# Need to adapt Alzheimer's disease criteria in Latin America

**DOI:** 10.1002/alz.14289

**Published:** 2024-10-06

**Authors:** Nilton Custodio, Ricardo Allegri, Francisco Lopera, Paulo Caramelli

**Affiliations:** ^1^ Unidad de diagnóstico de deterioro cognitivo y prevención de demencia Instituto Peruano de Neurociencias Lima Peru; ^2^ Servicio de Neurología Cognitiva Instituto Neurológico Fleni Buenos Aires Argentina; ^3^ Grupo de Neurociencias de Antioquia Facultad de Medicina Universidad de Antioquia Medellin Colombia; ^4^ Behavioral and Cognitive Neurology Unit Faculdade de Medicina Universidade Federal de Minas Gerais Belo Horizonte (MG) Brazil

The demographic transition to an increase in life expectancy is occurring faster in Latin America and Caribbean (LAC) countries than in high‐income countries (HIC), leading to a higher prevalence of chronic and neurodegenerative disorders, such as dementia. Indeed, a recent systematic review and meta‐analyses reported that the prevalence of all‐cause dementia in LAC is 9% among individuals aged 65+ years, with a higher prevalence among women, lower‐educated individuals, and in rural areas.^1^ LAC countries are diverse in terms of culture, socioeconomic status, education, and ethnicity. Socioeconomic and educational disparities explain the inequalities on minorities,^2^ mainly due to a lack of awareness, the stigma associated with aging and dementia, insufficient training of health professionals, and limited availability of assessment tools, especially for primary care, besides the fact that most LAC countries do not have a national dementia plan.^3^

Recently, the Alzheimer's Association (AA) updated the criteria for diagnosis and staging of Alzheimer's disease (AD).^4^ These criteria are fundamentally based on biological aspects of the disease that can be detected by specific biomarkers measured by positron emission tomography (PET), cerebrospinal fluid (CSF), or blood. We foresee that the main problems in adapting the revised criteria to LAC lie in (1) the categorization of imaging biomarkers, as we shall not be able to consider core 1 (Aβ proteinopathy) and core 2 (AD Tau proteinopathy) criteria, and (2) biological staging as a criterion for treatment because few research centers will be able to complete the imaging criteria due to the absence of PET. Nowadays, the use of PET is currently recommended by various international AD guidelines,^5^ its application in LAC countries remains restricted to a few centers in Argentina, Brazil, Colombia,^6^ and Uruguay.

Magnetic resonance imaging (MRI) is an essential tool that allows structural analysis of brain regions, which assists the diagnostic workup and follow‐up and constitutes a biomarker of neurodegeneration and vascular copathology.^4^ In this sense, the LatAm‐FINGERS study uses a complex harmonization procedure to ensure external harmonization with other neuroimaging cohorts (e.g., ADNI), and internal harmonization among centers to enable data sharing across databases while considering regional feasibility.^7^ Similarly, the Multi partner Consortium to explore dementia research in Latin America (ReDLat) applying different postrecording harmonization has been utilized for understanding neurodegeneration and for the development of multimodal markers in Argentina, Brazil, Chile, Colombia, Mexico, and Peru.^8^ Only Argentina, Brazil, and Colombia have conducted studies using CSF biomarkers in different samples, principally carriers of *PSEN1* mutation and noncarriers^6^ and isolated reports combining MRI with biomarkers on CSF or blood sample,^9^ but we lack information on the best candidates, local normative values, cutoff points, and the time‐of‐day conditions in which they should be collected. Some plasma p‐tau assays have demonstrated very good clinical performance in clinical trials and observational studies as a standalone biomarker,^4^ although with scarce evidence in community‐based studies. The first and largest study ever carried out in a South American population (Peru and Bolivia) investigating AD diagnosis by plasma p‐tau217 found an AUC of 68%–82% depending on the different adjusted models, validating the blood biomarker's modest performances in a non‐White population.^10^ However, the values are lower concerning other populations,^4^ so it is necessary to have a larger number of samples and different populations of LAC.

While it is true, the AA Workgroup emphasizes that these criteria are for research, especially for clinical trials, and of debatable application in clinical practice. In the past 3 years, the United States Food and Drug Administration (FDA) approved anti‐amyloid agents, demonstrating a reduction in brain amyloid and in the rates of cognitive and functional decline after 18 months of treatment. For future adequate use of these anti‐amyloid agents in clinical practice in LAC, specialists need to harmonize these new diagnostic criteria based on local availability of biomarkers. So, researchers and clinicians specialized in dementia management need to investigate how these criteria apply in the regional context. We anticipate that the increasing availability of plasma biomarkers will make these criteria more widely deployable within LAC region where PET‐ and CSF‐based biomarkers may not be readily available. However, the diagnostic performance of these plasma biomarkers across different populations in LAC needs to be investigated. In order to reduce the gap between LAC and HIC, we need to develop a LAC working group on local recommendations for biological diagnosis based on knowledge of local resources, development of regional collaborative networks, and knowledge transfer and exchange of protocols and procedures. New biological criteria for diagnosis AD in LAC need to take into consideration regional barriers in LAC and proposals to implement it (Table 1).

**TABLE 1:** Barriers and proposals to improve the implementation of the use of biomarkers for the diagnosis of Alzheimer's disease in Latin America and the Caribbean

Context	Biomarker	Barriers	Proposals to implementation
Research	Blood	Available in a few research centers for ADRD. Lack of cost‐effective evidence in primary care. Lack of evidence of efficiency in indigenous populations of LAC. Absence of guidelines to suggest its administration as screening tools to select better candidates for CSF or PET studies.	Stimulate the formation of basic and advanced research centers in ADRD to process blood‐based biomarkers. Stimulate the application of blood‐based biomarkers in epidemiological studies or primary care centers. Stimulate blood‐based biomarkers research in diverse populations in LAC. Investigate the potential value of blood‐based biomarkers as screening tools to improve the selection of candidates for CSF or PET studies.
	CSF	Myths about the procedure. Processing and transport of samples to laboratories in the USA. Lack of validation of reference values. Lack of evaluation of probable confounding factors in the LAC population for the correct interpretation of results.	Increase knowledge about lumbar puncture. Stimulate processing of CSF samples in local centers. Stimulate validation of local reference values. Control confounding factors in processed samples.
	Amyloid PET	Available in only two countries (Argentina and Uruguay). Absence of local cyclotrons. 3. Lack of commercial availability markers.	Coordinate with local governments the implementation of PET. Improve processes for radioactive materials produced in local cyclotrons. Encourage the use of commercial availability markers.
	Tau PET	Available only in Argentina.	Encourage its implementation in other LAC centers.
Clinical practice	Blood	High costs for families (out‐pockets expensive from USD 650 to 1000). Processing and transporting samples to laboratories in other countries. Lack of validation of reference values. 4. Lack of guidelines to suggest its administration as screening tools to select better candidates for CSF or PET.	1 and 2. Centralize and improve costs with a provider within LAC. 3. Stimulate the validation of local reference values. 4. LAC consensus for the correct application of guidelines for the use of blood‐based biomarkers and then PET.
	CSF	High costs (out‐pockets expensive from USD 350 to 800) and a long time to receive the results (> 60 days). Processing and transporting samples to laboratories in other countries. Lack of validation of reference values. 4. Lack of evaluation of probable confounding factors in the LAC population for the correct interpretation of results.	1 and 2. Centralize and improve costs with a supplier within LAC. 3. Encourage validation of local reference values. 4. Control confounding factors in processed samples.
	Amyloid PET	High costs for families (out‐pockets expensive from USD 1660 to 2800). Absence of local cutoff values to establish the diagnosis. 3. Lack of knowledge of possible confounding factors in the LAC population for the correct interpretation of results.	Negotiate with local suppliers more affordable prices. Encourage validation of local cutoff values. 3. Control confounding factors in processed samples.
	Tau PET	NA	Encourage its implementation in some centers.

Abbreviations: ADRD, Alzheimer's disease and related disorders; CSF, cerebrospinal fluid; LAC, Latin American and Caribbean; NA, not available; PET, positron emission tomography; USD, United States dollars.

## CONFLICT OF INTEREST STATEMENT

The authors declare no conflicts of interest. Author disclosures are available in the Supporting Information.

## Supporting information



Supporting Information
